# Microinjection Molding of Out-of-Plane Bistable Mechanisms

**DOI:** 10.3390/mi11020155

**Published:** 2020-01-30

**Authors:** Wook-Bae Kim, Sol-Yi Han

**Affiliations:** 1 Department of Mechanical Design Engineering, Korea Polytechnic University, Siheung 15073, Korea; 2 R&D Devision, Eosystem, Incheon 22829, Korea; hansolyi307@gmail.com

**Keywords:** bistable mechanism, V-beam structure, compliant mechanism, microinjection molding, out-of-plane

## Abstract

We present a novel fabrication technique of a miniaturized out-of-plane compliant bistable mechanism (OBM) by microinjection molding (MM) and assembling. OBMs are mostly in-plane monolithic devices containing delicate elastic elements fabricated in metal, plastic, or by a microelectromechanical system (MEMS) process. The proposed technique is based on stacking two out-of-plane V-beam structures obtained by mold fabrication and MM of thermoplastic polyacetal resin (POM) and joining their centers and outer frames to construct a double V-beam structure. A copper alloy mold insert was machined with the sectional dimensions of the V-beam cavities. Next, the insert was re-machined to reduce dimensional errors caused by part shrinkage. The V-beam structure was injection-molded at a high temperature. Gradually elongated short-shots were obtained by increasing pressure, showing the symmetrical melt filling through the V-beam cavities. The as-molded structure was buckled elastically by an external-force load but showed a monostable behavior because of a higher unconstrained buckling mode. The double V-beam device assembled with two single-molded structures shows clear bistability. The experimental force-displacement curve of the molded structure is presented for examination. This work can potentially contribute to the fabrication of architected materials with periodic assembly of the plastic bistable mechanism for diverse functionalities, such as energy absorption and shape morphing.

## 1. Introduction

Bistable mechanisms (BMs) are defined as mechanical systems that exhibit two stable states in two different positions [1,2]. A movable structure in BMs switches quickly from one position to another when a force is exerted beyond a threshold value and keeps a stable state even under small environmental disturbances without an external power. Due to their unique force-displacement behavior, BMs have been used for diverse applications such as switches, latches, valves, clamps, actuators, robotics, and energy harvesting [3,4,5,6,7,8].

Unlike the traditional latch-lock, a compliant mechanism produces bistability by storing and releasing strain energy from its flexible structural members during movement. In many microsystems, a compliant bistable (CB) mechanism has been widely used because devices are easily fabricated in monolithic form with no conventional mechanical elements to be assembled, such as bearing, pin and spring. The simplest elastic-buckling CB structure is a bent beam or plate created by simply holding a business card between two fingers and folding it. It snaps laterally and stays this way after applying an external force to its surface. 

To create this buckling behavior on a small-scale structure, the axial load necessary may result from the fabrication process residual stress or from direct compression of the corresponding beam with a come drive or electrothermal actuator conveniently placed on it [9,10]. A more simplified method to obtain buckling motion without residual stress and stress loading is to use double curved or V-shaped beams clamped together at their centers [11,12,13].

In microsystems, BMs use mainly silicon-based materials, bringing benefits such as compliant structure precision, process reproducibility, and high mechanical strength. However, silicon materials, as compared to polymer and metal, suffer from compliant-motion limited displacement due to their small yield strain (Ratio of strength to Young’s modulus). Metal offers good properties to BMs for its strength and toughness. However, it has limited miniaturization and a high manufacturing cost, the latter due to elaborate fabrication processes such as wire electric discharge machining (WEDM) and precision cutting. On the other hand, MEMS fabrication, using metal and silicon materials, need a complex process to produce three-dimensional structures [14,15].

Plastic is a good candidate for compliant mechanisms (CMs) including bistable systems due to its flexibility and cost effectiveness. Its yield strain is in the order of 5% to 10%, higher than that of metal, silicon, and ceramics (in metals it is close to 0.1% [16]). Although plastic is less resistant and its behavior unpredictable, it is essentially useful for structures with low stiffness and large displacement. 

An injection molding process (IM) can offer the freedom to create three-dimensional structures WEDM and MEMS cannot provide; it offers mass production at a relatively low cost, making it suitable for micro-CMs. Nevertheless, there are typical problems with monolithic CMs injection molding. It is a challenge to apply it to an elastic member like a slender beam, as the small cavity thickness caused by short shot causes the quick cooling of the molten polymer. This is common in the MM process for various high precision micro-components such as micro-gear, microfluidic devices, and microlens arrays [17,18]. For more than ten years, there has been a growing interest in MM, especially on the influence of replication-fidelity process parameters such as melting and molding temperature, injection speed and holding pressure. High settings of these parameters generally give a positive effect on the replication of micro-features. In addition, the influence degree of key parameters could depend on part layout, materials, and mold roughness and coating features [19,20,21,22].

Plastic micro-cantilever beams have been injection-molded into micro-chemical and biomedical sensors, atomic force microscopes, and micro-springs. Injection molding of micro-cantilever or micro-bridge structures were the subject of several studies that seek to understand the mechanism of microinjection molding [23,24,25,26,27,28]. A few of those reveal a high length-to-thickness ratio in beam structures in the order of tens or even over a hundred. This is possible using a mold temperature controller that switches to a high setting in a melt-filling stage and to low in a cooling one. Except for a micro-cantilever, there has been little research on good quality, high length-to-thickness ratio beams for specific function; that is, fabrication and testing of compliant BMs using MM, and as far as the authors know, it has hardly ever been researched. 

In this study, stacking two single V-beam structures to construct the double V-beam structure is proposed as a new method to produce OBMs. We first designed out-of-plane BMs using 150 µm thick vertically inclined slender beams, forming V-shape structures. We examined the part layout for IM success of a single V-beam structure and observed the force-displacement behavior of the stacked double V-beam structure dimension through a finite element analysis (FEA). Then, IM was used to create the designed single mechanisms and molding parameters were set to obtain parts without defects, such as flashes and short shots. Dimensional errors caused by shrinkage were compensated by re-machining mold inserts. Finally, two molded V-beam structures were stacked up to construct a double V-beam device to produce bistable motion and testing was completed by measuring force-displacement relationships for every molded part.

## 2. Design of Double V-Beam Bistable Mechanism 

### 2.1. Beam-Based Bistable Mechanisms (BM)

**Figure 1:**
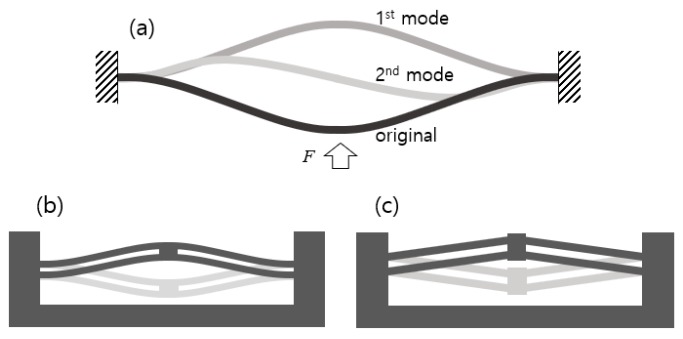
Compliant bistable mechanisms: (**a**) First and second post-buckling states of a pre-compressed beam; (**b**) curved parallel beams; (**c**) V-shaped parallel beams.

The V-beam BM was inspired by a stress-free, as-fabricated curved beam with an initial first buckling-mode shape. Figure 1a shows the single cosine curved beam that was made rectilinear with fixed ends. When a lateral force is applied, bistable motion is produced by post-buckling phenomena and the beam takes the shape of the first deflection mode. In this single-curved beam, however, the buckled beam in the first deflection mode moves back to its original shape when the lateral force is removed. Qiu et al. presented two criteria for bistable motion to occur, based on the buckling mode analysis of a curved beam: (1) the ratio of the curve height (apex height) to the beam thickness is large and (2) the asymmetric second mode must be constrained because the force with the second mode acts in the direction to original state of the beam [2]. To obtain stable bistability with a single-curved beam, compressive internal stress needs to be induced. Generally, two or several parallel beams connected at their centers are used to constrain the secondary mode and produce robust bistability, since adding axial loads would complicate a system, as shown in Figure 1b. The straight parallel V-beam structure in Figure 1c was manufactured using in-plane processes such as surface micromachining and WEDM. 

Fabrication of three-dimensional compliant members with force transfer and/or out-of-plane displacement is generally challenging, especially in miniaturized devices, leading to the design of complex 2.5D structures. Substrate out-of-plane BMs are very useful in various micro-systems. Beam buckling and bimorph effect of in-plane structure multi-materials have been used for out-of-plane motion, but in general, the creation of three-dimensional structure of at a micro level is quietly complex.

**Figure 2:**
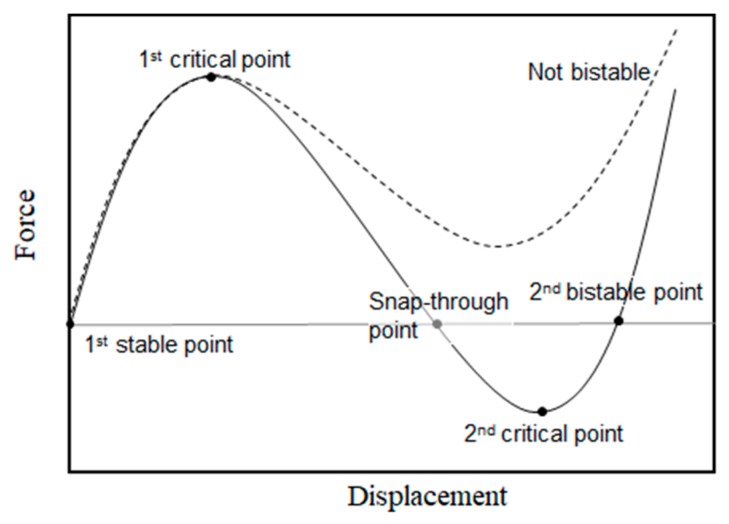
Force-displacement relation of a V-beam structure.

The V-beam bistable behavior can be expressed as the relationship between force and displacement, as shown in Figure 2. The first stable point is when the force does not have an effect, and as it increases to a maximum, it reaches the first critical-buckling force. After this point, we observe two different behaviors. In a bistable system, over the negative stiffness region, the beam reaches a snap-through point where the force starts to act opposite to the first stable point and quickly shifts to the second bistable point, passing a minimum value, the second critical-buckling force. At the second bistable point, the V-beam remains bent without the effect of an external force. In a non-bistable system, the beam never reaches the negative force, even as the displacement increases. The beam moves back to original position when the force is eliminated.

Force-displacement modelling and the resulting bistability predictability of pre-shaped beam structures were performed by the numerical solution for the differential equations of a post-buckling beam as well as the buckling mode analysis which can consider multiple (symmetric and asymmetric) buckling modes [2,12,13,15,29]. When the asymmetric mode can be constrained like in the double V-beam structure, the non-linear static FEA is also used for the force-displacement behavior [30,31,32,33]. 

### 2.2. Design Consideration for Injection Molding (IM) of Single V-Beam and Bistability

In the design stage, moldability and bistability should be considered simultaneously for successful fabrication. Essential features of compliant microsystem bodies are thin elastic beam segments. While beam slenderness is required, the attainable thickness-to-length ratio is limited because of its increased flow resistance of thermoplastic melt through cavities during a melt-filling stage. Consequently, the molded structure needs to be designed based on realistic data. In the author’s previous studies, a cantilever 50 µm thick and 2 mm long was made using a two-stage mold containing a cavity of beams formed along the parting plane with thermoplastic polystyrene (PS) and polyoxymethylene (POM) resin, using a mold temperature controller [23]. A longer beam could be moldable, although severe flashes appeared unavoidably on its edge along the parting plane, and low strength caused beam deformation during ejection. Beam thickness was set to 150 µm, and other parameters such as length and V-beam inclined angles were determined from nonlinear FEA, which evaluates the given structure and POM material force–displacement relationship (elastic modulus: 2570 MPa, yield strength: 62 MPa).

As described in Section 2.1, double centrally clamped beams are required with a high apex height to bean thickness ratio to achieve the bistable snap-through behavior. However, direct injection molding of double V-beams aligned in out-of-plane direction is considerably challenging because undercuts appear between two beams, preventing their ejection from the mold. Therefore, we designed a single V-beam structure as a molded part first and analyzed the force-displacement relation using nonlinear structural static FEA (ANSYS workbench 19, ANSYS, Inc., Canonsburg, PA, USA) for two single V-beams structures that were stacked and bonded into double V-beam structures.

**Figure 3:**
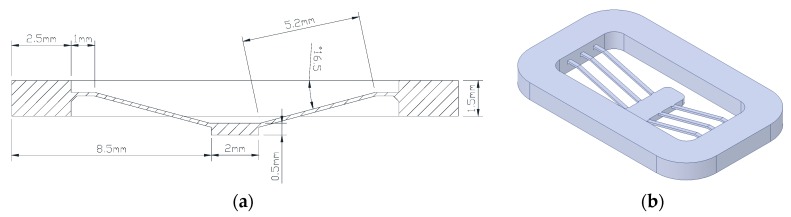
Design of single V-beam structures as mold part: (**a**) Cross section drawing; (**b**) 3D model.

Figure 3a shows a sectional drawing of a designed bistable structure. Two symmetrically inclined beams of 5.2 mm long form a V-beam with a central shuttle. They connect to a rectangular main frame 1.5 mm thick through a 1-mm-long horizontal beam segment, including lower taper. The latter is for smooth thermoplastic melt flow from thick to thin and to lower pressure loss and residual stress after solidification. The beam width in the ground direction was designed with two values: 0.2 mm and 0.3 mm and was 0.15 mm thick. The whole BM consisted of three parallel V-beams having the same center shuttle, fixed to the main frame (18.8 mm × 11.5 mm × 1.5 mm), as shown in Figure 3b. 

**Figure 4:**
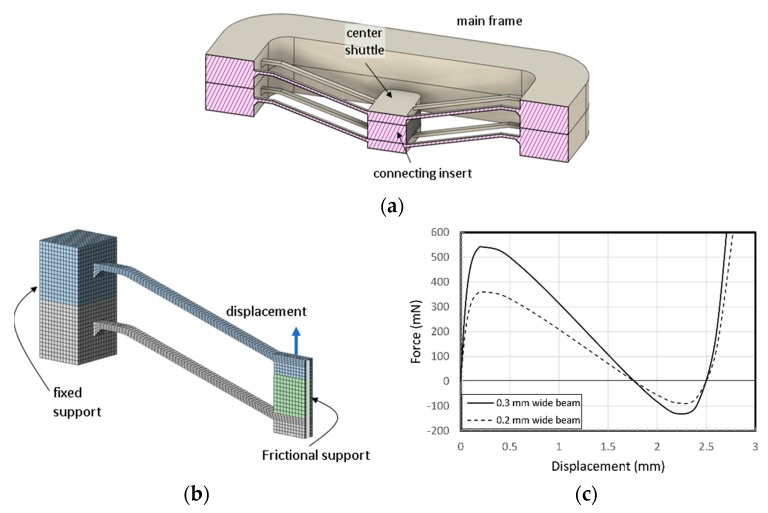
OBM based on V-beam structure stacking: (**a**) 3D sectional view; (**b**) FE model with boundary conditions; (**c**) simulated static force-displacement curves.

Figure 4a presents a three-dimensional cross section of the two-stacked model shown in Figure 3b. A connecting insert is placed between two center shuttles and put in bonded contact with the faces of two center shuttles (the insert, the same POM as the structure here, is replaced with glue, as described later in Section 4.2). Two main frames were also put in boned contact with each other. A half-symmetry model of one double V-beam structure was built with the boundary conditions, as shown in Figure 4b. Hexahedral meshing was applied to the model and the total number of elements were 8190 for the 0.3 mm wide V-beam model and 7410 for the 0.2 mm model, respectively. The displacement of 2.8 mm was applied with 24 loading steps (0.02 mm successive increments to 0.2 mm, the 0.2 mm increments up to 2.8 mm). The calculated displacement as a function of applied force for the designed BM with three double V-beams is shown in Figure 4c. It is known that it has enough bistability.

## 3. Fabrication

### 3.1. Layout and Tooling

**Figure 5:**
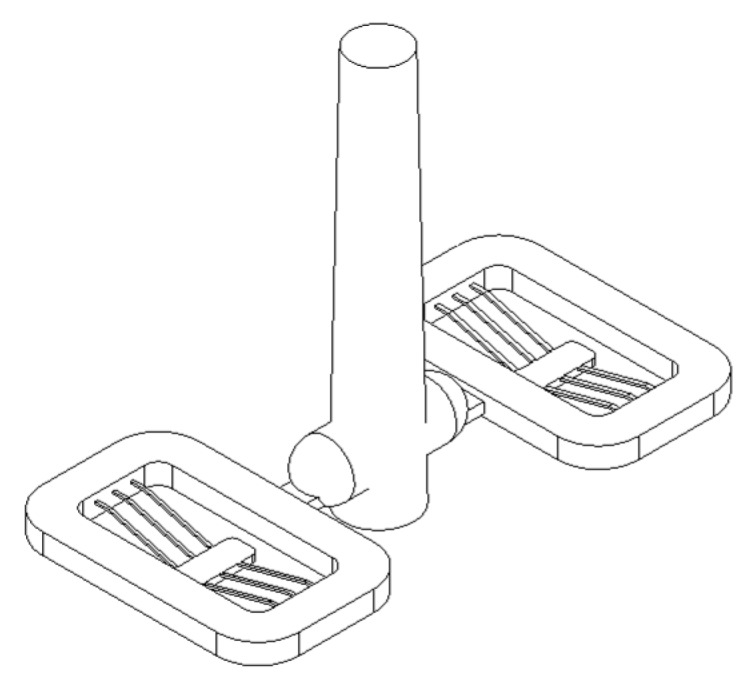
Melt delivery system.

A melt delivery system was designed using a symmetric two-cavity layout, as shown in Figure 5, considering the filling pattern of thermoplastic melt in mold cavities. Flow, cooling and solidification different in melt delivery can cause non-uniform material properties of the molded part. Gates (width 2 mm, thickness 0.8 mm, land 1 mm) were placed at the center of the long side of the main frame so that the beams on both sides of the center shuttle were subjected to the same conditions, as weld lines are formed inside where the fronts of two melt flows meet. 

**Figure 6:**
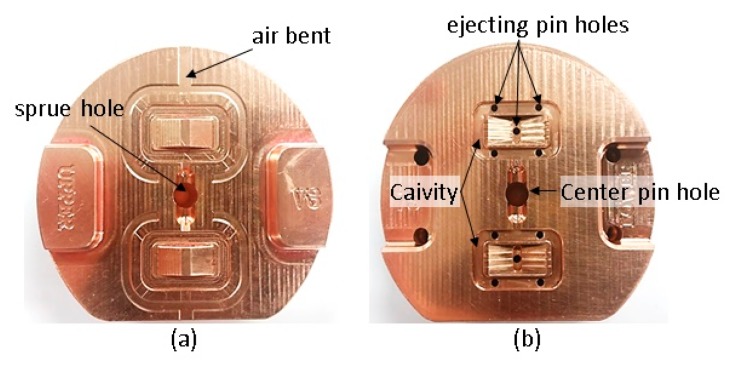
Machined mold insert: (**a**) fixed side; (**b**) movable side.

The runner diameter is 4 mm and its length (from the sprue center to the gate) is 5.8 mm. An alloy of beryllium copper was used for the unibody insert, containing the melt delivery system and the part cavities, and fabricated using a high-speed milling machine. The parting line was created along the top edge of the V-beam and the cavities formed in a movable sided mold insert that is shown in Figure 6. Upper and lower cavities were for a 0.3 mm and a 0.2 mm wide beam, respectively. 

**Figure 7:**
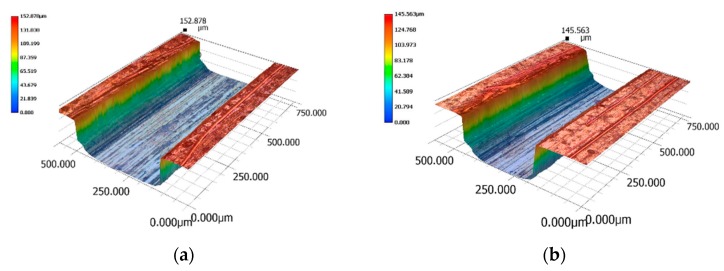
Three dimensional digital microscope images of machined cavities for 0.3 mm (**a**) and 0.2 mm (**b**) wide beam.

A 3-D digital microscope (Keyence VHX-900F, Keyence Corporation, Osaka, Japan) was used to measure the micro-cavities dimensions and the results are shown in Figure 7. The cavity sections were close to trapezoidal form and the corners were round at the bottom edge, presumably caused by tool deflection [28,29]. The beam cavities widths at the midpoints of the thickness were 330.5 µm and 238.1 µm respectively, and their thicknesses about 144 µm and 142 µm. Even though dimensional errors in beam cavities are directly associated to the molded beams and their stiffness, their effect is negligible as the moment of inertia for each beam changes less than 3%. The surface roughness on the cavity bottom is Rms 0.25 µm. The ejector pins contact four points on the rectangular frame and one pin was pushed up the center shuttle.

### 3.2. Material and Injection Molding

The polymer material used in IM is a POM (Lucel N109, melt flow index: 9 g/10 min, supplied by LG Chemistry, Seoul, Korea), the semi-crystalline engineering plastic. POM has been widely used in MM research due to its processability, such as low viscosity and thermal stability. POM is also known for its high-tensile strength, rigidity, high fatigue resistance, natural lubricity, and environmental stability. An IM machine with a vertical single plunger and a diameter of 16 mm (LS-30, Canon Electronics, Tokyo, Japan) was used. Its maximum injection speed is 75 mm/s and clamp force is 29 kN. 

In order to fill the cavities completely through the melt delivery system in Figure 4, high settings for injection speed and holding pressure are required. A fast mold temperature control is also indispensable, in which heater rods and chilled air are applied into the mold insert of Figure 5 [23,26].

The process parameter values applied for a successfully molded BM are shown in Table 1.

**Figure 8:**
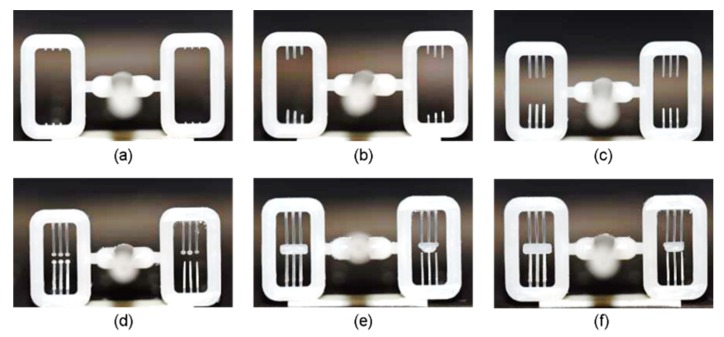
Short shots obtained during melt filling stage, by increasing injection pressure with other factors fixed (Injection pressure of (**a**) 20, (**b**) 25, (**c**) 30, (**d**) 45, (**e**) 55, and (**f**) 65 MPa).

Figure 8 shows the short shots obtained by increasing the injection pressure from 20 MPa to 65 MPa, filling the micro-cavity by the stagnant pressure in the main cavity. The left side of the central sprue contains the micro-cavities of the 0.3 mm wide beam and the right side contains those of the 0.2 mm wide beam. As shown in Figure 8a, filling begins in all micro-cavities on both sides and its length increases symmetrically but slightly faster in the left side due to its higher width, described in Figure 8b–d. Figure 8e shows the two-melt fronts meeting at the central shuttle in the left-side cavity, but the weld lines are out of center in the right one, which might have been caused by asymmetrical-cavity dimensional error. Such unbalanced filling gives rise to asymmetrical deformation of the molded V-beam when a force acts on the center shuttle after. 

**Figure 9:**
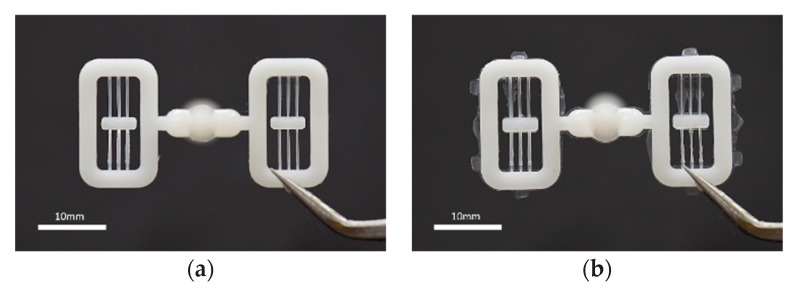
Molded V-beam structures in good quality (**a**) and with flashes (**b**).

Flash is a common defect in IM caused not only by an excessive cavity pressure over the nominal clamping force, but plastic melt high flowability due to high mold or melt temperature. Especially when molding a micro-beam, flash can easily occur under high settings of mold/melt temperature and injection speed [24,27]. In our experiment, severe flash was encountered when the mold temperature controller increased to 140 °C at filling, as shown in Figure 9. It formed even at 130 °C but this amount of flash did not adversely affect the deformation. To obtain a part with no defects, successful molding condition should be set carefully because the range is very narrow between short shot and flash occurrence.

### 3.3. Shrinkage and Shape Error Compensation

**Figure 10:**
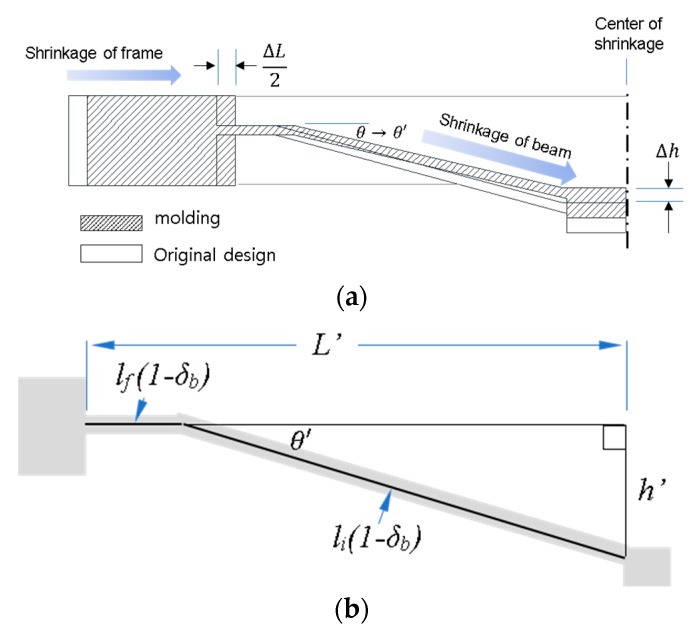
Model of shrinkage for mold compensation: (**a**) shrinkage and change of geometry (half of molded part); (**b**) notations of dimension for shrunk structure.

The elimination of thermal shrinkage of molded parts during cooling and ejection is likely a challenge even when melt fills completely the micro-cavities. Thermal shrinkage causes V-beam geometric errors from the original design, which needs to be minimized as to not affect the bistability. As described in Figure 10a, it was assumed that shrinkage occurs towards the center of the part, and main frame and beams longitudinal shrinkages give rise to vertical position changes of the center shuttle as well as of the inclined beam angles (Δ*h* and Δ*θ = θ − θ′*). It is also assumed that the shrinkage ratios are different between the beam and the main frame because a thinner beam cools much faster. 

The length of the main frame was measured with a profile projector (PJ-A3000, Mitutoyo, Kawasaki, Japan) taking the average of six molded samples (The distance is measured between the edges of the fixed end of a beam) and used to determine the shrinkage ratio along the same length in the mold insert. Instead of measuring the V-beam length directly due to it difficulty, a height gauge was used to measure the vertical deviation of the center shuttle. Taking the shrinkage ratio in the longitudinal direction of the micro-beam *δb*, the right-angled triangle relation can be applied to find *δb*, as shown in Figure 10b. The original lengths of the horizontal and inclined sections of the beam are lf and ls. The horizontally shrunken length of the main frame in the region of the beam is *L′*, and *h′* is the vertical position of the center shuttle, which can be determined by measuring the gap between the two horizontal planes formed by the main frame and the center shuttle. The inclined angle of the beam in the molded part is *θ′*.

Using the Pythagorean Theorem, beam shrinkage ratios are shown in Table 2. The ratio for the beam was 1.08%, smaller than that of the main frame at 1.78%. The general shrinkage ratio from the supplier is 1.8%–2.1%, close to the main frame value. We modified the dimensions to reduce a large error in *h*, *θ*, and *L*. Those for the compensated mold and those resulting under the same conditions used for the first molding experiment are shown in Table 2. The shrinkage is similar in both the main frame and the beam. The error values compared to the original design were clearly reduced. 

## 4. Experiment

### 4.1. Setup for Measurement of Force-Displacement Behavior 

**Figure 11:**
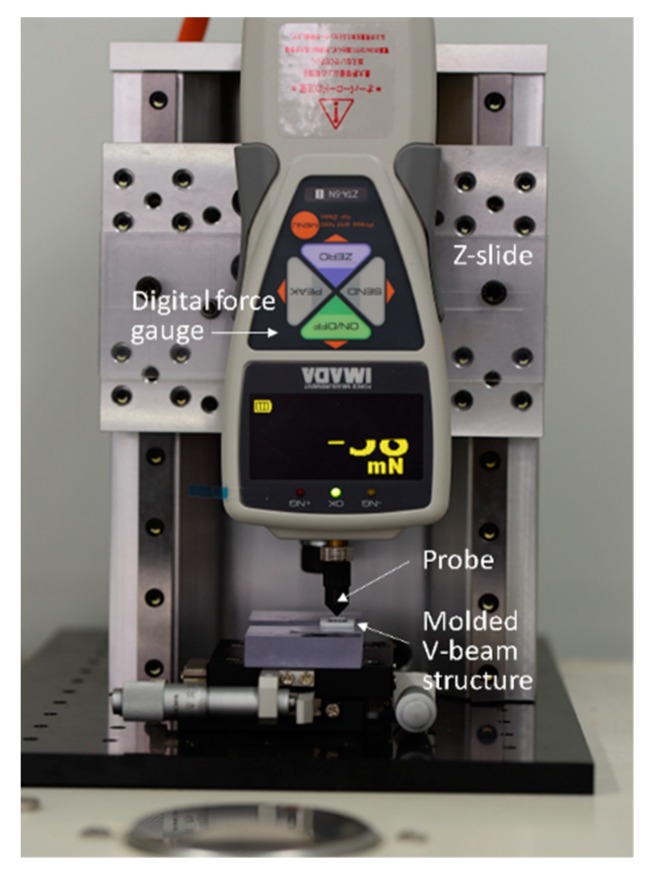
Setup of the force-displacement measurement experiment.

To obtain the relationship between the driving force and the center-shuttle vertical displacement in molded V-beam structures, a digital force gauge (ZTA-5N, Imada, force resolution 1 mN, maximum force capacity 5 N) was fixed on a linear stage so its probe can press the shuttle central point, as shown in Figure 11. Both molded main-frame sides were attached to a block with a XY-stage-mounted groove. After touching the center of the shuttle, the probe moved down further 2.8 mm while deforming the V-beam. Force values of 10 as-molded samples were measured at intervals of 1 mm from the point of contact.

### 4.2. Force–Displacement Relationship of Molded V-beam Structures

**Figure 12:**
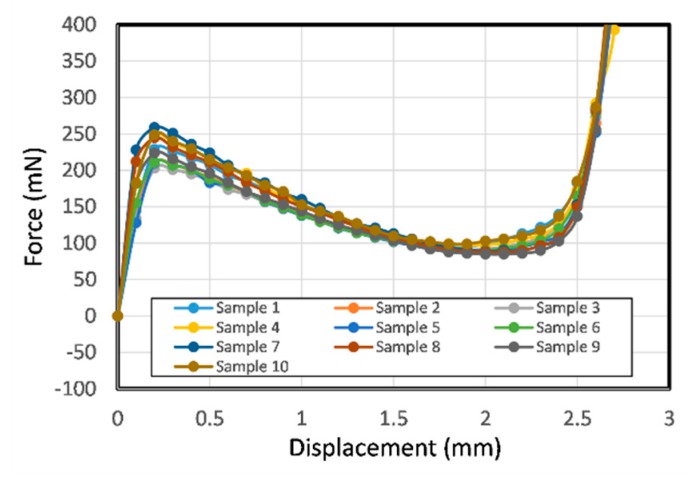
Experimental force–displacement relationship for ten molded samples of single V-beam structure.

Figure 12 shows the measured force-displacement curves for the ten as-molded single V-beam structures. In the curves, the force increases with the displacement in the early region and after the peak point, the measured forces show negative stiffness, though they do not become negative, failing to produce bistable motion as previously discussed in Section 2.1. This is because the unconstrained, S-shaped second mode was produced, thus preventing snap-through bistable motion.

**Figure 13:**
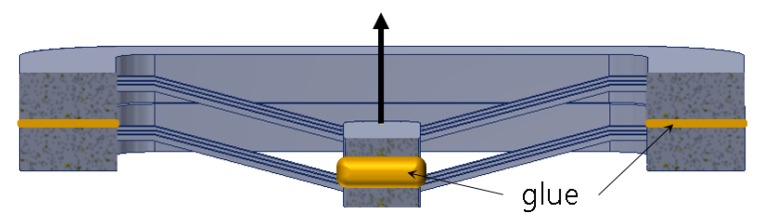
Double V-beam structure made by stacking up two molded single V-beam parts.

We constructed a double V-beam structure OBM by stacking up two molded V-beams structures using hot melt adhesive to glue the top and bottom surfaces of the main frames and the center shuttle together, as shown in Figure 13.

**Figure 14:**
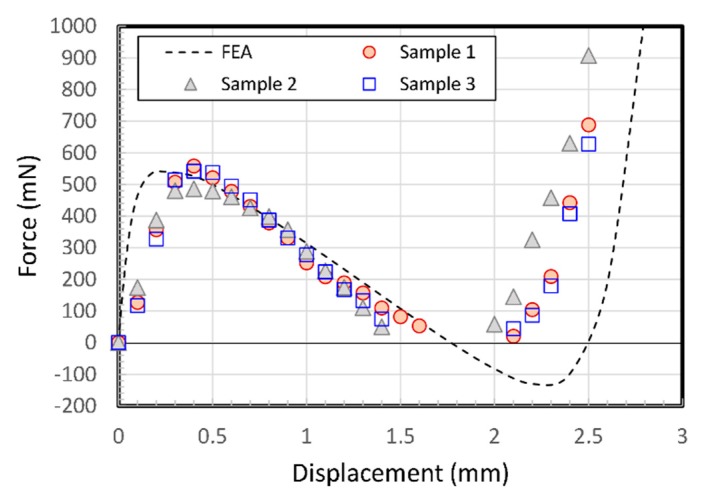
Experimental force-displacement relationship for three assembled double V-beam samples.

Figure 14 shows a graph of its experimental behavior from three fabricated samples. The peak force doubles with respect to the displacement of a single V-beam structure. All assembled-double-V-beam structures show clear snap-through behavior and bistability. 

**Figure 15:**
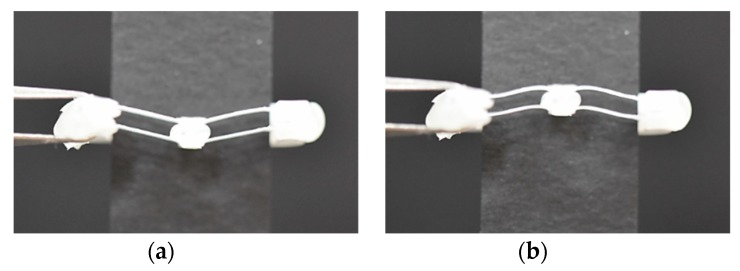
Photograph of the structure (front main frame segment is cut off and a black paper strip covers the rear frame to make the micro-beams clearly visible) in (**a**) the original position and (**b**) the second stable position after snap-through.

Negative stiffness leads to zero at displacements of 1.5–2 mm, caused by quick snap-through action. The negative force data did not appear on the graph because the shuttle was detached from the probe when the snapping action occurred. The experimental results show some discrepancies with the numerical solution, especially in the early displacement and after the snap-through point, which may be due to soft adhesive layer connecting two parts and relative position errors. If assembling the two structures was more improved, the behavior would be more predictable. The bistability of the assembled double V-beam structure is shown in Figure 15, in which the V-beams are at two stable points. 

## 5. Conclusions

Our study highlights that miniaturized plastic BMs based on compliant V-beam-like structures can be fabricated with good quality by MM technology and stacking up two V-beam structures. In the MM process, thermoplastic melt fills completely the micro-beam cavities of the high length-to-thickness ratio over 40 using high-temperature conditions with design for injection-molding. If bistable motion can be extended to multiple dimensions with balanced kinetic characteristics through mechanism design research and/or automatic motion can be produced by smart or stimuli responsive designs using like shape memory polymers, the high productivity and low cost characteristics of the injection-molding process could eventually lead to its application in various microsystems [34,35,36,37]. 

Recently, architected materials, defined as materials whose effective properties result from their ordered microarchitecture rather than their intrinsic material properties have been attracting considerable interest [38,39,40,41,42,43]. Fast advances in additive manufacturing technologies have enabled precise fabrication of new types of complex architected materials. Periodic BMs with symmetric slender beams can be applied to reversible energy-absorbing and tunable morphological changing architected materials, opening a range of new functionalities. We hope that a bottom-up assembly process can be used as the fabrication technique for three-dimensional functional materials along with the advanced additive manufacturing techniques. Furthermore, BMs fabricated by MM have enough potential to be used as a unit cell if a modular design plan and the optimized mechanical response are provided, together with the proper part design of the assembly [44,45].

## Figures and Tables

**Table 1:** Parameter settings for complete filling.

Process Parameters	Value
Injection speed (mm/s)	75
Injection pressure (MPa)	75
Pressure holding time (s)	4
Melt temperature (°C)	220
Mold temperature (°C)	130 (filling), 100 (ejecting)

**Table 2:** Dimensions of mold and molded parts of the original and the compensated mold, whose errors were obtained by comparing original design dimensions.

Design	Geometric Variable	Mold	Part	Shrinkage (%)	Error (%)
Original	*L* (mm)	5.83	5.726	1.78	−1.78
	*l_f_* (mm)	1.0	0.99	1.08	−1
	*l_i_* (mm)	5.0	4.95		−1
	*h* (mm)	1.294	1.175	–	−9.1
	*θ* (°)	15.0	13.74	–	−8.4
Compensation	*L* (mm)	5.99	5.869	2.02	0.6
	*l_f_* (mm)	1	0.99	1.01	−1
	*l_i_* (mm)	5.2	5.15		3
	*h* (mm)	1.45	1.34	–	3.6
	*θ* (°)	16.4	15.08	–	0.6
